# Unraveling the role of computed tomography derived body composition metrics on anastomotic leakages rates in rectal cancer surgery: A protocol for a systematic review and meta-analysis

**DOI:** 10.1371/journal.pone.0307606

**Published:** 2024-07-24

**Authors:** Mark Broekman, Charlotte M. S. Genders, Ritchie T. J. Geitenbeek, Klaas Havenga, Schelto Kruijff, Joost M. Klaase, Alain R. Viddeleer, Esther C. J. Consten

**Affiliations:** 1 Department of Surgery, Groningen University Medical Center, University of Groningen, Groningen, The Netherlands; 2 Department of Surgery, Meander Medical Center, Amersfoort, The Netherlands; 3 Department of Radiology, Medical Imaging Center, University Medical Centre Groningen, University of Groningen, Groningen, The Netherlands; Hacettepe University: Hacettepe Universitesi, TÜRKIYE

## Abstract

**Introduction:**

Anastomotic leakage is a major concern following total mesorectal excision for rectal cancer, affecting oncological outcomes, morbidity an treatment costs. Body composition has been suggested to influence anastomotic leakage rates. However, literature on how body composition impact anastomotic leakage rates is conflicting. This systematic review aims to evaluate the role of computed tomography derived body composition metrics on anastomotic leakage rates in rectal cancer patients.

**Methods:**

Databases PubMed/MEDLINE, Cochrane Library, web of science, and EMBASE, will be systematically searched for papers from January 2010 onwards. Study selection, data collection and quality assessment will be independently performed by three research fellows. Outcomes described in three or more studies will be included in the meta-analysis. The Q-test and I2 statistic will be used to assess statistical heterogeneity between studies. Publication bias will be examined by visual inspection of funnel plots and quantified by Egger’s test. Sensitivity analyses will be conducted to examine the robustness of the meta-analysis. Reporting of the findings will be in line with the Preferred Reporting Items for Systematic Review and Meta-Analyses (PRISMA) guidelines.

**Conclusions:**

This systematic review will synthesize the current evidence and will identify knowledge gaps. Results of the systematic review will aid health care professional in pre-operative decision making and will be distributed through a peer-reviewed publication and presentation of results at international meetings.

**Trial registration:**

PROSPERO protocol number: CRD42023471537.

## Introduction

Rectal cancer is a major global health concern, with approximately 732.000 new cases diagnosed worldwide in 2020 [1]. The introduction of the total mesorectal excision (TME) principle by Heald et al. in 1982 revolutionized the surgical treatment of rectal carcinoma. This procedure, often preceded by (chemo)radiotherapy, improved long-term local recurrence-free and overall survival rates and has since been widely adopted as the standard surgical treatment for rectal cancer [2–4]. However, even with advances in surgical techniques and care, anastomotic leakage (AL) remains a serious complication that can occur in up to 20% of rectal cancer patients undergoing a restorative resection [5]. AL is reported to negatively affect oncological outcomes, morbidity, and increase treatment costs [6–8].

The pathogenesis of AL is multifactorial, and includes patient characteristics, tumour features, and surgical factors [9–12]. Given the complex causality, correctly identifying patients at risk for AL remains challenging despite numerous attempts [13, 14]. Recently, body composition has been suggested to affect AL rates [15, 16]. Body composition is a dynamic and modifiable entity that refers to the different tissue components within the body, including muscle mass and adipose tissue [17, 18]. Body composition assessment can easily be performed using a variety of imaging modalities, which are part of the routine workup for rectal cancer surgery [18–20]. Among these imaging techniques, computed tomography (CT) is deemed the most appropriate for body composition assessment [21].

Some studies have suggested that certain body composition metrics may be more useful in predicting AL risk than traditional risk factors, like obesity and sarcopenia [22–25]. These body composition metrics offer a better reflection of the patients’ metabolic condition, inflammatory status, and the specific technical challenges associated with surgery in the narrow pelvis [26–31]. By understanding how body composition affects AL, healthcare professionals can make a more informed decisions regarding patient preparation, thereby mitigating the risk of this complication. Nevertheless, conflicting findings have been reported by various studies, warranting a comprehensive review of the available evidence [32, 33]. Therefore, this systematic review aims to evaluate the role of computed tomography (CT) derived body composition metrics on AL rates in rectal cancer patients.

## Methods

### Registration details

This protocol is written in line with the Preferred Reporting Items for Systematic Review and Meta-Analysis Protocols guidelines (S1). The protocol is registered in the International Prospective register of systematic reviews (PROSPERO) (protocol number: CRD42023471537).

### Information sources and search strategy

A comprehensive systematic search will be conducted across several databases, including PubMed/MEDLINE, EMBASE, Scopus, Web of Science, and Cochrane Library. The literature search will be conducted using a pre-defined search string involving a combination of medical subject headings (MeSH) and standard search headings related to body composition, AL, and rectal cancer. Additionally, truncation and wildcard symbols will be used to capture spelling and word endings variations. Boolean operators will be employed to combine the search terms effectively. The initial search string will be developed for PubMed and adapted to fit other databases. To ensure the quality of the search string, the search string will be built with the help of a team of experienced researchers, including subject experts and a professional medical librarian experienced in searching for systematic reviews (SII).

The "related article" function in PubMed and a review of the included studies’ reference lists will be used to identify potentially relevant articles. Additionally, different clinical trial databases (i.e., World Health Organisation (WHO) Registry Network (including ClinicalTrials.gov), PROSPERO, EMBASE) will be screened.

### Eligibility criteria

The study eligibility criteria were defined in accordance with the Population/Participants, Intervention, Comparison, Outcome, and Study design (PICOS) framework. Population: adult (≥18 years old) patients with rectal cancer undergoing rectal cancer surgery. Intervention: the measurement of body composition parameters derived from CT scanning. We are interested in reviewing all muscle- and fat related body composition values, including but not limited to visceral fat area or index, subcutaneous fat area or index, skeletal muscle area or index, and psoas muscle area or index. Studies using other methods than CT to measure body composition parameters, such as magnetic resonance imaging (MRI), will be excluded. Outcome: the primary outcome of this systematic review will be AL rates. As the definition of AL may vary among individual studies, no pre-defined definition will be imposed. The ‘comparison’ heading from the PICOS framework is not applicable for this review as no comparators will be assessed.

Studies will be included if they are published in English, French, German, and Spanish. Studies will be excluded if they meet the following criteria: (1) Studies that are not primary research, including reviews; (conference) abstracts, commentaries, letters, editorials, case series, and case reports; (2) studies published before 2010; (3) studies including less than 10 patients; (4) animal studies or in vitro studies; (5) duplicate studies or multiple publications derived from the same sample size and finally (6) studies without full text available.

Considering the substantial advancements in the treatment of rectal cancer, studies published before the year 2010 will be excluded. In case of duplicate studies or multiple publications derived from the same sample size, the article with the largest sample size will be included in the review. Finally, if relevant articles in other languages are identified, these will be provided in list form as an appendix.

### Data management

The systematic review results will be uploaded to EndNote 21, a reference management tool widely recommended for handling duplicates in systematic reviews. Duplicates will be identified using the built-in ‘Find Duplicate’ tool in EndNote 21. Duplicates will be removed after assessing the identified duplicates on a case-by-case basis. Subsequently, the duplicate free results from the literature search will be uploaded to Rayyan QCRI. This is a web-based software management program that helps facilitate the screening and study selection process of authors of systematic reviews. Abstracts and full-text articles will be uploaded as PDF files.

### Selection process

Two independent review authors (MB and CG) will conduct title and abstract screening to identify potentially eligible records based on predefined criteria. Each article will be assigned a score (“not relevant”, “potentially relevant”, and “relevant”) based on the predefined eligibility criteria. Potentially relevant studies will undergo full-text screening for final inclusion by two independent review authors (MB, CG). Discrepancies will be resolved through discussion involving six additional authors, all of whom have expertise in the surgical treatment of rectal cancer (RG, KH, SK, JK, AV and EC). At this stage of the selection process, reasons excluding “potentially relevant” and “relevant” studies will be recorded and added as an appendix in the review. To illustrate the selection process, a flow diagram will be made using the PRISMA 2009 Flow Diagram.

### Data collection process

Following study selection, the included studies will be uploaded to CADIMA, an open excess software tool, facilitating the documentation of systematic reviews. A standardized data extraction form will be developed using the built-in data extraction tool in CADIMA. Data will be extracted from included eligible studies using this standardized data extraction form. Two review authors (MB, CG) will independently extract data from the included studies. To increase the consistency of data extraction between reviewers, instructions on using the standardized extraction form will be given before the start of data extraction. Disagreements between review authors will be resolved through discussion involving six additional authors, all of whom have expertise in the surgical treatment of rectal cancer (RG, KH, SK, JK, AV and EC). When the primary studies lack sufficient data for meta-analysis or specifics on treatment details, or outcomes of interest are missing, the study’s corresponding authors will be requested for additional data. A reminder e-mail will be sent up to three times over the duration of one month.

### Data items

The following PICOS related data will be extracted from eligible studies: (1) characteristics of study population, including number of patients, gender, age, ethnicity, height, weight, Body Mass Index, ASA classification, albumin, hemoglobin, history of abdominal surgery, history of smoking, diabetes mellitus; characteristics of disease, including cT/cN/cM stage, neoadjuvant therapy, tumor types, rectal cancer definition, distance from the anorectal junction, tumor location; (3) characteristics of surgery, including surgical technique, type of procedure, surgeon experience, operating time, anastomotic technique, height of the anastomosis to the ARJ, number of staples, use of fluorescence, deviating stoma creation, splenic flexure mobilization, ostomy creation, ostomy reversal, blood loss, intraoperative complications, conversion, bowel preparation, drain placement and transanal bowel drainage; (4) main findings related to body composition, including the body composition parameters reported with their definition, metrics, and imaging modality; (5) outcomes used, including AL definition, AL diagnosis, AL rates, type of AL, post-operative complications other than AL, Clavien Dindo classification, length of hospital stay, ICU stay, readmissions, re-interventions, local recurrence, systematic recurrence, disease free survival and overall survival. Furthermore, the following study-related outcomes will be collected: reference and title details, including first author, journal, year of publication, country, study design, study period, follow-up, funding received, and conflicts of interest. If needed, means of outcomes will be independently approximated from figures in the reports and checked for accuracy by a second review author. Different reporting outcomes will be evaluated for inclusion on a case-by-case basis.

### Risk of bias in individual studies

All studies will be assessed using the Cochrane Risk of Bias Tool for randomized controlled trials or the Newcastle-Ottawa scale (NOS) for non-randomized studies. The Cochrane Risk of Bias Tool consists of seven domains representing potential sources of bias. All domains will be assessed and receive a score of “low risk,” “high risk,” or “unclear risk”. The NOS consists of three domains representing key features of study quality. Based on these domains, all studies will receive a score ranging from 0 to 9, with higher scores indicating higher quality and a lower risk of bias. The quality of the included studies will be appraised by two independent review authors (MB, CG). Disagreement will be resolved through discussion with six additional authors (RG, KH, SK, JK, AV and EC) until a consensus is reached. A graphic or table representation of potential bias for each study will be presented.

### Statistical analysis

Statistical analysis will be performed using R statistical software. Categorical variables reported as numbers and percentages will be analysed using the χ2 test. Continuous data will be analysed using the Analysis of Variance/Kruskal-Wallis test. Outcomes described in three or more studies will be included in the meta-analysis. Odds ratios and 95% CI will be calculated to estimate the association between binary factors and AL. Within and between studies, variation of heterogeneity will be assessed by testing Cochran’s Q-statistic. The Q-test and I2 statistic will be used to assess statistical heterogeneity between studies. A significant Q statistic (p < 0.05) or a large I2 value (>50%) indicates substantial heterogeneity. If a difference in statistical heterogeneity is detected, a random-effect model will be applied to pool the effect size across studies. The overall effect size will be calculated using the DerSimonian and Laird methods. Otherwise, a fixed-model will be used. Publication bias will be examined by visual inspection of funnel plots and quantified by Egger’s test. Sensitivity analyses will be conducted to examine the robustness of the meta-analysis by identifying potential sources of heterogeneity based on the risk of bias. Sensitivity analyses will consist of, but are not limited to comparison of prospective studies versus retrospective studies, clinical AL versus non-clinical AL, CT versus other imaging modalities and different follow-up periods. The level of significance will be set at p < 0.05.

### Meta-bias(es)

Reporting bias amongst studies will be assessed by comparing outcomes reported in the published protocol (if available) with those reported in the published journal article. Additionally, small sample bias will be assessed by comparing the fixed effect estimate against the random effects model.

### Confidence in cumulative evidence

The Grading of Recommendations Assessment, Development and Evaluation working group approach (GRADE) will be used to assess the confidence in the cumulative evidence. The GRADE-tool will be utilized to evaluate all outcomes based on the risk of bias, consistency, directness, precision, and publication bias. Additional domains will be developed, if necessary, to assess specific outcomes. The quality of evidence will be reported as high (The authors have a high level of confidence that the true effect is similar to the estimated effect), moderate (The authors believe that the true effect is likely to be close to the estimated effect), low (The true effect may significantly differ from the estimated effect), or very low (The true effect is expected to markedly differ from the estimated effect). If meta-analysis is performed, the GRADE approach will also assess the strength of evidence for studies excluded from meta-analysis.

### Ethics

According to Dutch law, this study is classified as a non-WMO (Medical Research Involving Human Subjects Act) study, as this systematic review does not involve the collection of individual patient data.

### Study timeline

Articles will be screened and selected from 1 March 2024 till 1 April 2024. Data will be collected, analysed and assessed for risk of bias from 1 May 2024 till 1 June 2024. drafting of the final manuscript will be done from 1 June 2024 till 1 August 2024.

## Discussion

### Strength and limitation of study

- The literature search for this systematic review will be performed in line with the, with the assistance of senior medical librarian.
- To minimize the impact of missing data, authors will be contacted and asked to provide additional information if necessary. The systematic review will be performed with support of experts in the field of rectal cancer surgery and body composition
- Difference in the definition of anastomotic leakage may hinder comparison between studies.

### Dissemination plans

The results of the review will be widely distributed through peer-reviewed publication, and presentations at related congresses.

### Amendments

The date of amendment and rationale for deviation will be provided in case of substantial protocol amendments or deviations.

## Supporting information

S1 File PRISMA checklist. (DOCX)

S2 File Search syntax. (DOCX)
