# Supplementary material for: Unraveling the role of computed tomography derived body composition metrics on anastomotic leakages rates in rectal cancer surgery: A protocol for a systematic review and meta-analysis
Source: PLoS One. 2024 Jul 24;19(7):e0307606. doi: 10.1371/journal.pone.0307606 (PMC11268673; doi:10.1371/journal.pone.0307606)
Supplement: S2 File — (DOCX) [file pone.0307606.s002.docx]

**Supplementary File II**

**PubMed**

("Colorectal Neoplasms"[Mesh] OR ((colorect*[tiab] OR colon*[tiab] OR rect*[tiab] OR anus*[tiab]) AND (cancer*[tiab] OR neoplasm*[tiab] OR tumo*[tiab] OR carcinoma*[tiab])))

AND

("Body Composition"[Mesh] OR "Psoas Muscles"[Mesh] OR "Sarcopenia"[Mesh] OR "Muscular Atrophy"[Mesh] OR "Muscle, Skeletal"[Mesh] OR "Body Constitution"[Mesh] OR body composit*[tiab] OR body mass*[tiab] OR body constitut*[tiab] OR body weight*[tiab] OR Fat measur*[tiab] OR fat distribut*[tiab] OR Body measur*[tiab] OR Visceral Adipos*[tiab] OR Visceral fat area*[tiab] OR Visceral obesit*[tiab] OR Muscle measur*[tiab] OR Muscle mass*[tiab] OR Muscle index*[tiab] OR Muscle area*[tiab] OR Muscle attenuation*[tiab] OR muscle densit*[tiab] OR muscle qualit*[tiab] OR muscle quantit*[tiab] OR muscle area*[tiab] OR muscle atroph*[tiab] OR Psoas densit*[tiab] OR Psoas muscle*[tiab] OR Psoas measur*[tiab] OR myopenia*[tiab] OR Myosteatos*[tiab] OR Adipos*[tiab] OR Sarcopen*[tiab])

AND

("Postoperative Complications"[Mesh] OR "Anastomosis, Surgical"[Mesh] OR “Morbidity” [Mesh] anastomo*[tiab] OR complicat*[tiab] OR morbidit*[tiab])

**EMBASE**

('colorectal cancer'/exp OR ((colorect*:ab,ti,kw OR colon*:ab,ti,kw OR rect*:ab,ti,kw OR anus*:ab,ti,kw) AND (cancer*:ab,ti,kw OR neoplasm*:ab,ti,kw OR tumo*:ab,ti,kw OR carcinoma*:ab,ti,kw)))

AND

('body composition'/exp OR 'psoas muscle'/exp OR 'sarcopenia'/exp OR 'muscle atrophy'/exp OR 'skeletal muscle'/exp OR 'body constitution'/exp OR ‘body composit*’:ab,ti,kw OR ‘body mass*’:ab,ti,kw OR ‘body constitut*’:ab,ti,kw OR ‘body weight*’:ab,ti,kw OR ‘Fat measur*’:ab,ti,kw OR ‘fat distribut*’:ab,ti,kw OR ‘Body measur*’:ab,ti,kw OR ‘Visceral Adipos*’:ab,ti,kw OR ‘Visceral fat area*’:ab,ti,kw OR ‘Visceral obesit*’:ab,ti,kw OR ‘Muscle measur*’:ab,ti,kw OR ‘Muscle mass*’:ab,ti,kw OR ‘Muscle index*’:ab,ti,kw OR ‘Muscle area*’:ab,ti,kw OR ‘Muscle attenuation*’:ab,ti,kw OR ‘muscle densit*’:ab,ti,kw OR ‘muscle qualit*’:ab,ti,kw OR ‘muscle quantit*’:ab,ti,kw OR ‘muscle area’:ab,ti,kw OR ‘muscle atroph*’:ab,ti,kw OR ‘Psoas densit*’:ab,ti,kw OR ‘Psoas muscle*’:ab,ti,kw OR ‘Psoas measur*’:ab,ti,kw OR myopenia*:ab,ti,kw OR Myosteatos*:ab,ti,kw OR Adipos*:ab,ti,kw OR Sarcopen*:ab,ti,kw)

AND

('postoperative complication'/exp OR 'anastomosis'/exp OR ‘morbidity’/exp OR anastomo*:ab,ti,kw OR complicat*:ab,ti,kw OR morbidit*:ab,ti,kw) NOT 'conference abstract'/it

**Web of Science**

TS=((“colorect*” OR “colon*” OR “rect*” OR “anus*”) AND (“cancer*” OR “neoplasm*” OR “tumo*” OR “carcinoma*”))

AND

TS=(“body composit*” OR “body mass*” OR “body constitut*” OR “body weight*” OR “Fat measur*” OR “fat distribut*” OR “Body measur*” OR “Visceral Adipos*” OR “Visceral fat area*” OR “Visceral obesit*” OR “Muscle measur*” OR “Muscle mass*” OR “Muscle index*” OR “Muscle area*” OR “Muscle attenuation*” OR “muscle densit*” OR “muscle qualit*” OR “muscle quantit*” OR “muscle area*” OR “muscle atroph*” OR “Psoas densit*” OR “Psoas muscle*” OR “Psoas measur*” OR “myopenia*” OR “Myosteatos*” OR “Adipos*” OR “Sarcopen*”)

AND

TS=(“anastomo*” OR “complicat*” OR “morbidit*”)

**Cochrane library**

ID Search

#1 MeSH descriptor: [Colorectal Neoplasms] explode all trees

#2 (colorect*):ti,ab,kw

#3 (colon*):ti,ab,kw

#4 (rect*):ti,ab,kw

#5 (anus*):ti,ab,kw

#6 (cancer*):ti,ab,kw

#7 (neoplasm*):ti,ab,kw

#8 (tumo*):ti,ab,kw

#9 (carcinoma*):ti,ab,kw

#10 (#1 OR (( #2 OR #3 OR #4 OR #5) AND (#6 OR #7 OR #8 OR #9))

#11 MeSH descriptor: [Body Composition] explode all trees

#12 MeSH descriptor: [Psoas Muscles] explode all trees

#13 MeSH descriptor: [Sarcopenia] explode all trees

#14 MeSH descriptor: [Muscular Atrophy] explode all trees

#15 MeSH descriptor: [Muscle, Skeletal] explode all trees

#16 MeSH descriptor: [Body Constitution] explode all trees

#17 (body composit*):ti,ab,kw

#18 (body mass*):ti,ab,kw

#19 (body constitut*):ti,ab,kw

#20 (body weight*):ti,ab,kw

#21 (fat measur*):ti,ab,kw

#22 (fat distribut*):ti,ab,kw

#23 (body measur*):ti,ab,kw

#24 (visceral adipos*):ti,ab,kw

#25 (visceral fat area*):ti,ab,kw

#26 (visceral obesit*):ti,ab,kw

#27 (muscle measur*):ti,ab,kw

#28 (muscle mass*):ti,ab,kw

#29 (muscle index*):ti,ab,kw

#30 (muscle area*):ti,ab,kw

#31 (muscle attenuation*):ti,ab,kw

#32 (muscle densit*):ti,ab,kw

#33 (muscle qualit*):ti,ab,kw

#34 (muscle quantit*):ti,ab,kw

#35 (muscle area*):ti,ab,kw

#36 (muscle atroph*):ti,ab,kw

#37 (psoas densit*):ti,ab,kw

#38 (psoas muscle*):ti,ab,kw

#39 (psoas measur*):ti,ab,kw

#40 (myopenia*):ti,ab,kw

#41 (myosteatos*):ti,ab,kw

#42 (adipos*):ti,ab,kw

#43 (sarcopen*):ti,ab,kw

#44 (#11 OR #12 OR #13 OR #14 OR #15 OR #16 OR #17 OR #18 OR #19 OR #20 OR #21 OR #22 OR #23 OR #24 OR #25 OR #26 OR #27 OR #28 OR #29 OR #30 OR #31 OR #32 OR #33 OR #34 OR #35 OR #36 OR #37 OR #38 OR #39 OR #40 OR #41 OR #42 OR #43)

#45 MeSH descriptor: [Postoperative Complications] explode all trees

#46 MeSH descriptor: [Anastomosis, Surgical] explode all trees

#47 MeSH descriptor: [Morbidity] explode all trees

#48 (anastomo*):ti,ab,kw

#49 (complicat*):ti,ab,kw

#50 (morbidit*):ti,ab,kw

#51 (45 OR #46 OR 47 OR #48 OR #49 OR #50)

#52 #10 AND #44 AND 561

**Filters and limits**

Limits were imposed for date of publicatios. This review will include studies published between january 2010 and January 2024 in the search strategy
